# Electroacupuncture at PC6 (Neiguan) Improves Extracellular Signal-Regulated Kinase Signaling Pathways Through the Regulation of Neuroendocrine Cytokines in Myocardial Hypertrophic Rats

**DOI:** 10.1155/2012/792820

**Published:** 2011-08-24

**Authors:** Jia Li, Jing Li, Fengxia Liang, Yaqun Hong, Song Wu, Hongtu Tang, Hua Wang

**Affiliations:** ^1^Department of Acupuncture and Moxibustion, Hubei University of Chinese Medicine, Wuhan 430061, China; ^2^Department of Traditional Chinese Medicine, Union Hospital, Tongji Medical College, Huazhong University of Science & Technology, Wuhan 430022, China

## Abstract

Electroacupuncture (EA) therapy has been widely accepted as a useful therapeutic technique with low or no risk in the clinical prevention of cardiac hypertrophy. However, the signaling transduction mechanism underlying this effect remains unclear. The current study investigates the effects of EA on the signaling pathways of myocardial hypertrophy (MH) in rats. Up to 40 3-month-old Sprague-Dawley (SD) rats were randomly divided into normal, model, PC6 (Neiguan), and LI4 (Hegu) groups, with ten rats in each group. All the rats except for the normal group received 3 mg/kg·d of isoprinosine hydrochloride (ISO) injection into the back skin. The rats in the PC6 and LI4 groups received EA for 14 days. On the 15th day, electrocardiograms were recorded, and the ultrastructure of the myocardial cells was observed. The myocardial hypertrophy indices (MHIs), electrocardiograph (ECG), ultrastructure observation, levels of plasma angiotensin II (Ang II) and endothelin (ET), as well as protein expression of extracellular signal-regulated kinase (ERK), and phosphorylation extracellular signal regulating kinase (p-ERK) in the left ventricular myocardial tissue were measured. The results indicated that EA can improve cardiac function in MH rats by modulating upstream neuroendocrine cytokines that regulate the ERK signaling pathways.

## 1. Introduction

In long-lasting pathologic emergency cases, myocardial hypertrophy (MH) may lead to coronary heart disease, congestive heart failure, stroke, and so on and can easily cause patient death or sudden death [1]. On a cellular and molecular level, the pathogenesis of MH may divide into three links, namely, extracellular hypertrophic stimulation, intracellular signal transduction, and intranuclear gene transcriptional activation, and eventually trigger hypertrophic myocardial cell changes. The intracellular signaling pathway is the coupling link of exocellular stimulus and nuclear gene activation. Thus, this signaling pathway has been proven as a sally port for researching the pathogenesis of MH, with extensive prospects for its application [2].

Angiotensin II (Ang II) and endothelin (ET) play a central part in MH, myofibrosis cordis, and cardiovascular reconstruction process. Lines of evidence showed that Ang II could enhance the expression of immediate early gene IEG (c-fos, c-jun, c-myc, etc.), terminal myocardial cell genes (skeleton actin, atrial natriuretic polypeptide), and transforming growth factor *β* gene in rat myocardial cells, confirming that the upregulation of these genes induces ventricular hypertrophy in the rat model [3]. Borges et al. [4] revealed that Ang II could facilitate MH and cardiovascular reconstruction, depending mainly on local secretion and release. ET is polypeptide that causes vasoconstriction, with a slow, but long-lasting and wide effect [5]. ET-1 causes strong coronary arterial constriction and has positive chronotropic action and positive inotropic action; therefore, it plays an important part in myocardial infarction [6]. These two neuroendocrine cytokines play an essential role in MH and act as upstream factors of extracellular signal-regulated kinase (ERK) signaling pathways [7]. 

ERK plays a key role in MH reaction, including gene expression and protein synthesis increase [8], which is not only referred to myocardial signal transduction and growth, but also closely related to MH myocytes and apoptosis [9]. Recent animal studies indicate that ERK activation is a key factor in regulating cardiac hypertrophy [10]. As we know, ERK signaling pathways mediate extensive biological effects [11], involving cell proliferation, cell apoptosis, inflammatory responses, oxidative stress, and even influences of the form and evolution of tumors. Research has confirmed that ERK1/2 is closely related to MH and that ERK is activated through ERK cascade (Ras/Raf/MEK/ERK) [12]. Therefore, the ERK signaling pathway was chosen as the research pointcut to explore the influence of electroacupuncture (EA) on MH, as well as to provide evidence from basic experiments to clinical research.

Acupuncture, undeniably the most well-known complementary and alternative medical treatment, has been proven as one of the most popular therapies in the world. Multiple researches confirmed that acupuncture could play a stable hypotensive effect achieve improvement or normalization of contractile function and diastolic values, a decrease of energy loss, and reversal of MH [13]. It has been well documented that acupuncture can effectively improve symptoms of angina, palpitation, and so forth. and improve the left cardiac function in coronary heart disease patients to inhibit MH [14], but the signaling pathways mechanism of the remarkable transformation caused by the treatment remains unknown.

Neiguan (PC6), a classical and experimental acupuncture point, has been recorded in ancient Chinese medical literature for thousands years and is preferred effectively in treating cardiovascular disorders. We learned that acupuncture had a more positive effect in increasing the glucose metabolic level in a stroke-injured area in the brain than nonacupoint stimulus and blank controls. In the present study, a real acupoint Hegu (LI4) located near PC6 was selected as the control point to compare its therapeutic differences with PC6.

Acupuncture indeed improves the cardiac function in MH, and this effect may have a close relation with ERK signaling pathway through regulating the role of neuroendocrine cytokines. The aim of the current study is to investigate whether EA could mediate ERK signaling pathway through regulating Ang II and ET. Two acupoints, PC6 and LI4, were also compared to observe the distinctive difference between them. 

## 2. Materials and Methods

### 2.1. Animals and Model

Female Sprague-Dawley rats, 3-month-old, weighing 170–180 g, were randomly divided into four groups: normal, model, PC6, and LI4 groups, with ten rats in each group. The rats received free food and water under a controlled temperature (24 ± 1°C), with 12 hours of artificial light per day.

Following the method by Yin et al. [15], rats were continuously injected every morning with 3 mg/kg of isoprinosine hydrochloride (ISO) into the back skin for 14 days, while rats in normal group were treated with an equal volume of physiologic saline, and rats were observed for behavior. The experimental procedures were carried out in accordance with the guidelines for the care and use of Laboratory Animals published by National Institutes of Health of the United States.

### 2.2. EA

The PC6 rats were given improvised clothing and subjected to acupuncture on PC6 after an injection of ISO. The LI4 rats were treated in the same way, except that the acupoint was instead of LI4. The acupoints were referred from the “Map of the Experimental Animal Acupuncture Points,” formulated by the Experimental Acupuncture Institute of China Association of Acupuncture and Moxibustion. The acupuncture needle, 15 mm long and 0.3 mm in diameter, was penetrated 2-3 mm into the subcutis. The bilateral acupoints were interconnected with a Hans acupoint nerve stimulator (HANS) EA apparatus, and the following stimulus parameters were selected: continuous-wave at 2 Hz and 1 mA, for 20 min a day.

### 2.3. Electrocardiograph (ECG) Recording Electrode Resettlement

A BL-420 physiological function experiment system (Chengdu TME Technology Co, Ltd, China) was used to record the standard II-lead ECG. Negative and positive stainless steel electrodes were placed horizontally beneath the skin of the left and right forelimbs, and the reference electrode was placed beneath the skin of the right hind limb. Recording was performed at a resolution ratio of 500 nv/mv and a chart speed of 50 ms/div; 10 cardiac cycles were included into the calculation.

### 2.4. Ultrastructure Observation

The central ventricular muscle of the left ventricular free wall (LVFW) was chopped into 1 mm × 1 mm × 1 mm pieces, fixed in 2.5% glutaraldehyde for 48 h and 1% osmium tetrachloride for 1.5 h, dehydrated, embedded, sectioned, and examined under a Hitachi H-600 transmission electron microscopy (Japan's Hitachi Company, Japan).

### 2.5. Myocardial Hypertrophy Index (MHI)

The body weights of the rats, as well as those of their left ventricle and the whole heart, were recorded; then their MHI, left ventricular weight index (LVWI), and the heart weight index (HWI) were calculated.

### 2.6. Radioimmunoassay (RIA) Method

The plasma levels of Ang II and ET were determined with RIA in a full-automatic Gc-911 RIA counter (Zhong Jia Science and Technology Industry Company, China). At the end of the experiment, rats were executed, blood was collected, and plasma was frozen and stored until assayed. Plasma Ang II and ET activity was detected with an intrarun coefficient of variation under 10%; the interrun coefficient of variation under 15% (Beijing North Institute of Biological Technology, China). For analysis, we get log10-dose reference system into use.

### 2.7. Western Blotting

The expression of ERK and phosphorylation extracellular signal regulating kinase (p-ERK) in left ventricle tissue was measured with western blotting. About 100 mg left ventricular myocardial tissues was collected after being departed from heart on ice. The tissues were homogenized in a tissue lysis buffer as turn into lysates. Then the lysates were centrifuged at 12,000 g for 10 minutes, and the supernatant was transferred into another tube to be tested. Protein concentrations were determined using the BCA protein assay (Pierce). Equivalent amounts of protein (30 *μ*g/lane) were resolved electrophoretically by SDS-polyacrylamide gels (10%) and transferred onto PVDF membranes. Nonspecific reactivity was blocked in 5% skim milk in PBST (10 mM Tris-HCl, pH 7.5, 150 mM NaCl, 1% Tween-20) for 1 h at 6–8°C. Afterwards, membranes were incubated with ERK and P-ERK antibodies (1 : 200; Santa Cruz, Calif, USA) overnight at 4°C, and followed by secondary antibodies for 1 hour at room temperature. Protein was visualized by using the enhanced chemiluminescence system (ECL, Beyotime Institute of Biotechnology, Jiangsu, China). 

### 2.8. Statistical Analysis

Data were expressed as mean  ± SD and analyzed with one-way ANOVA using SPSS13.0 software. *P* < 0.05 was considered statistically significant.

## 3. Results

### 3.1. EA Modulated ECG after ISO Injection on Rats

**Figure 1:**
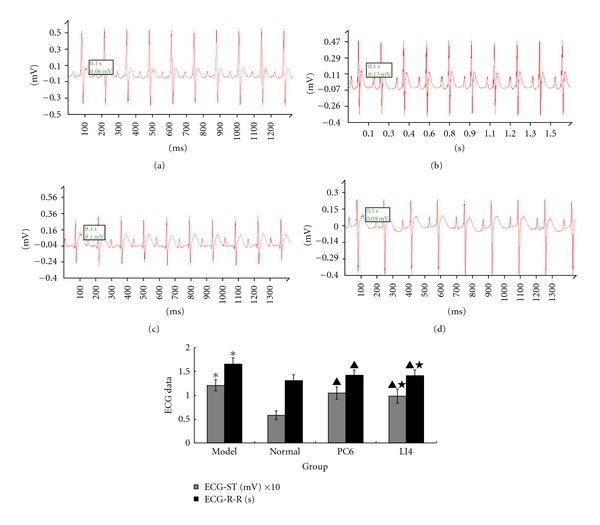
Comparison of the ECG-R-R and ECG-ST in rats for each group (*n* = 10 each group). ECG images are examples of four groups, involving 10 cardiac cycles in each figure. We can find out the changes in these pictures, normal group example ((a), R-R interval is 1.31 ms and ST amplitude is 0.06 mV), model group example ((b), R-R interval is 1.68 ms, and ST amplitude is 0.13 mV), PC6 group example ((c), R-R interval is 1.42 ms, and ST amplitude is 0.1 mV) and LI4 group example ((d), R-R interval is 1.42 ms, and ST amplitude is 0.09 mV). R-R interval prolonged and ST amplitude heightened in (b) while these two values diminished in (c, d). *P < 0.05 versus normal; ^▲^ *P* < 0.05 versus model; ^★^ *P* > 0.05 versus PC6.

Rats were observed for behavior, strong heartbeat, and tachypnea (above the standard 66–114 c.p.m., some of them could reach 150 c.p.m.) at 10 min after drug injection, and burnout sleepiness and weakness at 30 min after drug injection. ECG-R-R and ECG-ST were subjected to analysis (Figure 1). Compared with those in the normal group, the R-R interval and ST segment were significantly higher in the model group (*P* < 0.05). These two parameters tend to be normal in the PC6 and LI4 groups compared with the model group (*P* < 0.05). However, there was no statistically significant difference between the two EA groups (*P* > 0.05).

### 3.2. Effect of EA on Cardiac Cell Ultrastructure

**Figure 2:**
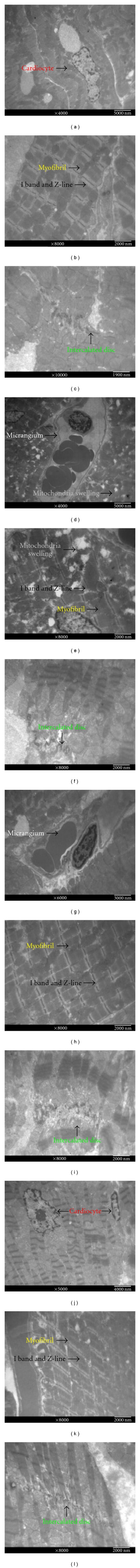
The ultrastructure of myocardium in rats for each group (*n* = 10 each group). Ultrastructure features in normal group ((a) ×4000, (b) ×8000, (c) ×10000), model group ((d) ×4000, (e) ×8000, (f) ×8000), PC6 group ((g) ×6000, (h) ×8000, (i) ×8000), and LI4 group ((j) ×5000, (k) ×8000, (l) ×8000). Cardiocyte and micrangium ((a, d, g, j), ×4000~6000), myofibril, I band, and Z-line ((b, e, h, k), ×8000), intercalated disc ((c,f,i, l), ×8000~10000). Some changes of the ultrastructure: mitochondria swelling ((d, e), cell apoptosis (j), endothelial cell interstitial hyperplasia (d), muscle plasma nets expansion (e), out-sync contraction of contraction band ((e, j), and intercalated disc deformation ((f, i, l). With reference to normal group, compared EA group with model group, EA significantly reduced cardiac muscular tissue damage.

MH showed a series of structural changes, including cardiomyocyte hypertrophy, interstitial connective tissue hyperplasia, and coronary circulation capillaries decrease [16]. In the current study, the rats in the model group mainly manifested six differences from the normal group (Figure 2), including mitochondria swelling, cell apoptosis, endothelial cell interstitial hyperplasia, muscle plasma nets expansion, out-sync contraction of contraction band, and intercalated disc deformation. With reference to normal group, compared EA group with model group, EA significantly reduced cardiac muscular tissue injury.

### 3.3. EA Treatment Downregulated MHI

**Figure 3:**
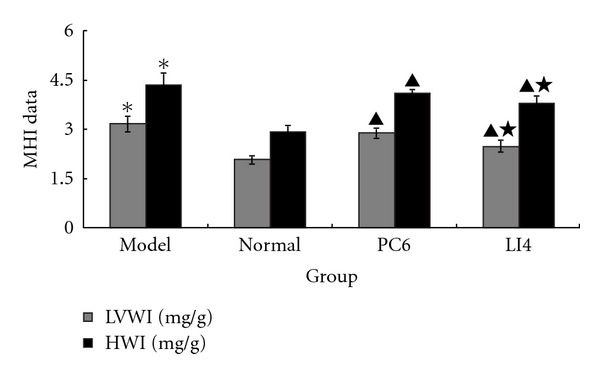
Effects of EA on LVWI, HWI in rats with MH induced by ISO (*n* = 10 each group). MHI: myocardial hypertrophy index, LVWI: left ventricular weight index, and HWI: heart weight index. LVWIs and HWIs in the model group were significantly higher compared with the model group. EA lowered the value, and LI4 was superior to PC6. *P < 0.01 versus normal; ^▲^ *P* < 0.05 versus model; ^★^ *P* < 0.05 versus PC6.

LVWI and HWI can reflex the MH level, providing an indication of the level of damage and the restoration of cardiac function. Compared with the normal group, the LVWIs and HWIs in the model group were significantly higher (*P* < 0.01). Compared with the model group, the two parameters were lower in the EA groups (*P* < 0.05). In addition, those in the LI4 group were superior to those in the PC6 group (*P* < 0.05), as shown in Figure 3.

### 3.4. EA Therapy Decreased Two Vasoconstrictors (Ang II and ET)

**Figure 4:**
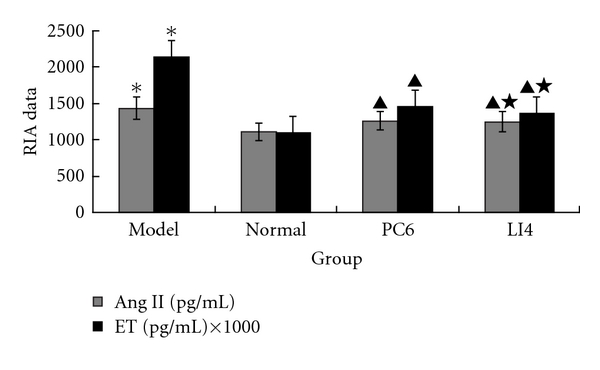
Effects of EA on the ratios of circulating Ang II, ET in rats for each group (*n* = 10 each group). The plasma Ang II, ET level of model group was significantly increased compared with normal group. EA decreased these cytokines, but there was no statistically significant difference between PC6 group and LI4 group. *P < 0.05 versus normal; ^▲^ *P* < 0.05 versus model; ^★^ *P* > 0.05 versus PC6.

Figure 4 shows that the Ang II and ET levels in the model group were significantly higher than in the normal group (*P* < 0.05) whereas these two parameters in EA groups were lower than those in the model group (*P* < 0.05). However, there was no statistically significant difference between these two EA groups (*P* > 0.05), as shown in Figure 4.

### 3.5. Effect of EA Downregulated ERK and p-ERK Protein Expression

**Figure 5:**
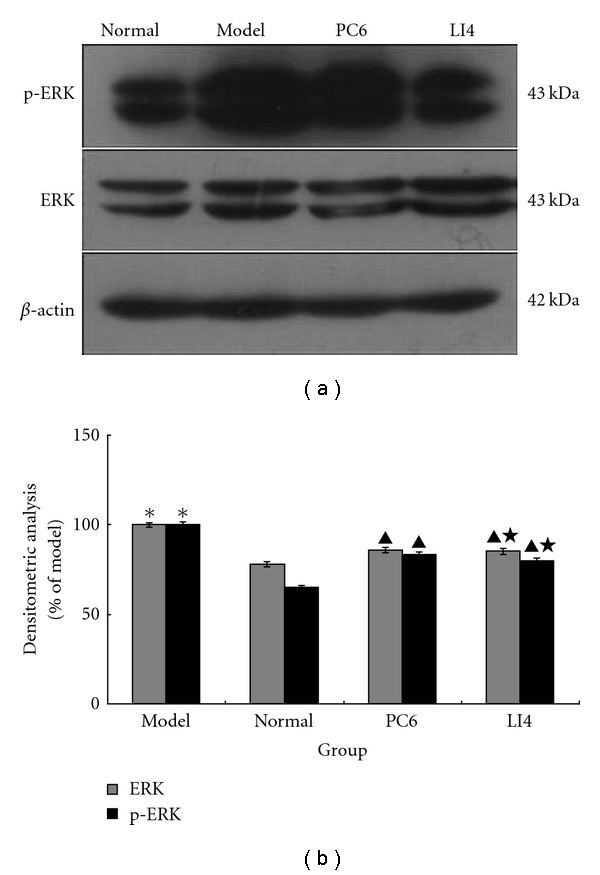
Effects of EA on the expression of ERK1/2 and p-ERK in cardiac muscular tissue in rats for each group (*n* = 10 each group). Western blotting was performed on protein extracts from left ventricular myocardial tissue. Quantitative analysis revealed a significant increase in model group while decrease in EA groups. However, there was no statistical significance difference between PC6 group and LI4 group. *P < 0.05 versus normal; ^▲^ *P* < 0.05 versus model; ^★^ *P* > 0.05 versus PC6.

The expression of ERK and p-ERK in the model group were significantly higher than that in the normal group (*P* < 0.05), whereas these two parameters in EA groups were lower than in the model group (*P* < 0.05). However, there was no statistically significant difference between the two EA groups (*P* > 0.05), as shown in Figure 5.

## 4. Discussion

The World Health Organization lists approximately 40 diseases wherein acupuncture treatment is effective [17]. Modern research has extensively analyzed the beneficial effects of acupuncture on the cardiovascular system [18]. Growing evidences showed that acupuncture effect is closely related to neural mechanism, and it has been well documented that PC6 can effectively improve symptoms of postoperative nausea, vomiting [19], labor pain [20], and obese diabetic [21] via nervous system. To the best of our knowledge, the current study is the first time to report that EA can improve MH by regulating the ERK signaling pathways through adjusting upstream neuroendocrine cytokines (Ang II and ET). The present study is based on neural endocrine system to discuss the role of EA on MH.

As classical and preferred acupoints in the long history of China, the “song of eight major points (Ba Zong Xue Ge)” [22] states that PC6 and LI4 as “for heart and chest, it is point Neiguan, for face and mouth, Hegu controls,” which can be combined with clinical practice. Generally, the use of PC6 in acupuncture is advisable for the treatment of symptoms of heart and chest diseases, such as palpitation, stuffy chest, angina, nausea, and vomiting. The use of LI4 in acupuncture is also advisable for the treatment of symptoms of such diseases as toothache, facial pain, headache, nasal obstruction, and eye redness. PC6 is often preferred over LI4 for its improved efficacy in treating cardiovascular diseases. However, the current study found that LI4 could have a better effect than PC6, especially with statistically significant difference in the MHI experiment. These findings may be different from those of previous researches. In recent studies, acupuncture induced a significant decrease in the LF/HF ratio and a significant increase in HF power; manual acupuncture at both LI4 and SP6 (Point Sanyinjiao) acupoints may play a role in the treatment of dysmenorrhea with autonomic nervous system involvement [23]. Li et al. [24] also found that acupuncture on LI4 or PC6 could regulate heart rate. This may provide exact evidence that LI4 is useful in regulating the heart rate. In addition, study of Nayak et al. suggested that electrostimulation application by point surface electrodes at LI4, ST36 (Zusanli), HT7 (Shenmen), and LR3 (Taichong) points performed sedation of critically ill patients in the intensive care unit [25] thus, it can be suggested that LI4 application in painful diseases is more extensive than PC6. However, this unexpected result was only observed once, and further evidence was not obtained in the current study. Hence, this will be investigated further in future studies.

In the following respect, this study stimulated the acupoints with low-current and low-frequency (1 mA, 2 Hz) EA. Recent research showed that low-frequency (2 Hz) EA activated many more somatic afferents than high-frequency stimulation such as 10 and 20 Hz. Ten minutes of stimulation by 2 Hz EA on healthy volunteers can evaluate in terms of heart rate variability, pulse rate variability, and skin conductance response [26]. Similarly, thirty minutes of low-current, low-frequency (0.3–0.5 mA, 2 Hz) significantly inhibited the gastric-cardiovascular pressor reflex, whereas a similar period of EA at 40 or 100 Hz did not alter the response [27]. Current topic was designed to discuss brain stem responses to different frequencies at pericardial acupoints located over the median nerve, and researchers discovered that premotor sympathetic cardiovascular neurons that receive convergent input from the splanchnic and median nerves during low-frequency EA and manual acupuncture were inhibited similarly for prolonged periods by low-frequency [28].

ECG is often regarded as the basic indicator for judging cardiac function. An increase in the QRS interval [29, 30] and a changing ST-T interval [31] provide an accurate diagnosis of MH. Considerable evidence documents that changes of the ST-segment is an effective indicator of the severity of myocardial ischemia [32]. The current study found that the R-R intervals were elongated and ST-T amplitudes were evidently increased in the model group and that EA could regulate the values. This indicates that EA on PC6 or LI4 can be medicative for MH.

Our experimental results showed that Ang II and ET participate in the formation of MH and that EA can improve MH by regulating the role of neuroendocrine-cytokines. The development of MH induced by hemodynamic overload is very likely initiated by mechanical stress. However, the involvement of growth promoting factors (e.g., TGF-b and VEGF), hormones (such as Ang II and ET-1), and cytokines (for instance CT-1) cannot be foreclosed [33]. As a main active metabolite of renin-angiotensin system, Ang II plays a key role in promoting MH and myocardial fibrosis [34]. Previous literature has shown that endogenous Ang II can enhance MH due to ISO in rats [35]. Ang II in cardiac interstitial tissue is produced in the transformation of chymase and has a cardioactive effect on myocardial and sympathetic nerve endings that regulate cardiac pressure chronotropic action and promote MH [36]. As the strongest vasoconstrictor yet discovered, ET participates in the pathogenesis of various cardiac diseases, such as myocardial ischemia and hypertension [37].

The mitogen-activated protein kinases (MAPKs) signal pathway, including extracellular signal-regulated kinase (ERK)1/2, c-Jun NH2-terminal kinase (JNK), and p38 kinase, has been recognized as a central mechanism underlying development of many types of cardiovascular disease, including cardiac failure and hypertrophy [38]. As the latest and the most important pathway, ERK is mainly activated by the Ras/Raf/MEK/ERK cascade. This pathway involves a module of four protein kinases, ERK, MEK, Raf, and Ras. As a low-molecular-weight GTPase, the small (21-kDa) guanine nucleotide-binding protein Ras plays a central role in the regulation of cell growth and division and it acts as upstream molecule to regulate raf [39]. There are three isozyme in Raf family, Raf-1, A-Raf, and B-Raf [40]. Raf-1 has been reported to be activated by Ras.GTP acts as a key protein kinase to activate dual protein kinase MEK [41]. MEK in turn activates ERKs by phosphorylating their threonine and tyrosine residues [42]. ERK exists in two isoforms in mammalian cells, ERK1 and ERK2. P-ERK is a homocysteine thiolactone that is eventually transported into nuclear and acts on downstream transcription factors (ELK, AP-1, NF-*κ*B, and etc.) and then regulates related protooncogenes (such as c-fos, c-myc, c-jun, jun-B, and Egr-1), which lead to MH. ERK is not only an important regulating factor in the nervous system and in cell division, but also essential for the signaling pathways of various cytokines (ET-1, NA, Ang II, etc.) that promote MH [43]. The ERK signaling pathways can be stimulated upon G protein coupled receptor occupation by binding of hormones (by binding of ET-1 [44] and Ang II [45]). The current study found that EA could inhibit the expression of ERK and p-ERK protein through mediating various cytokines to improve cardiac function in MH.

## 5. Conclusion

**Figure 6:**
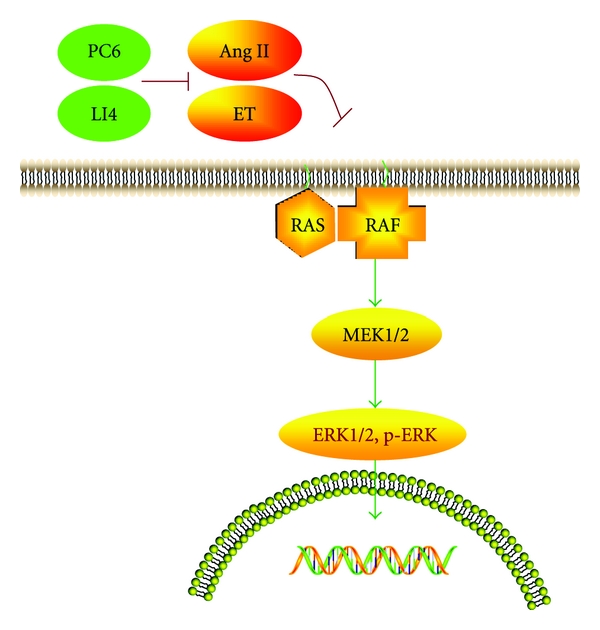
EA improves ERK signaling pathways through the regulation of neuroendocrine cytokines in myocardial hypertrophic rats. The proposed hypothetic mechanism of acupuncture affecting the heart acupuncture indeed improves the cardiac function in MH, and this effect has a close relation with ERK signaling pathway through regulating the role of neuroendocrine cytokines.

In summary, acupuncture improves cardiac function in MH, and this effect is closely related with the ERK signaling pathways through the regulation of neuroendocrine cytokines (Figure 6). EA stimulation of LI4 may be more effective than stimulation of PC6 in MH rats. This contrasts with previous research and could be a singular phenomenon that needs further verification.

##  Conflict of Interests

All authors manifest that there is no conflict of interests.
